# Role of temporal discounting in a conditional cash transfer (CCT) intervention to improve engagement in the prevention of mother-to-child transmission (PMTCT) cascade

**DOI:** 10.1186/s12889-021-10499-0

**Published:** 2021-03-10

**Authors:** Jessica Londeree Saleska, Abigail Norris Turner, Maria F. Gallo, Abigail Shoben, Bienvenu Kawende, Noro Lantoniaina Rosa Ravelomanana, Harsha Thirumurthy, Marcel Yotebieng

**Affiliations:** 1 Division of Epidemiology, Cunz Hall, The Ohio State University, College of Public Health, 1841 Neil Avenue, Columbus, OH 43210 USA; 2 The University of California Los Angeles, Global Center for Children and Families, Semel Institute for Neuroscience and Human Behavior, 10920 Wilshire Blvd, Los Angeles, CA 90024 USA; 3 Division of Infectious Disease, Doan Hall, The Ohio State University, College of Medicine, 410 W. 10th Avenue, Columbus, OH 43210 USA; 4 The University of Kinshasa, School of Public Health, Kinshasa, Democratic Republic of Congo; 5 Department of Medical Ethics and Health Policy, Perelman School of Medicine, University of Pennsylvania, Philadelphia, PA 19104 USA; 6 Center for Health Incentives and Behavioral Economics, University of Pennsylvania, Philadelphia, USA; 7 Department of Medicine, Division of General Internal Medicine, Albert Einstein College of Medicine, 3300 Kossuth Ave, Bronx, NY 10467 USA

**Keywords:** Temporal discounting, HIV, Prevention of mother-to-child transmission of HIV (PMTCT), PMTCT Cascade, Intertemporal preferences

## Abstract

**Background:**

Temporal discounting, the tendency of individuals to discount future costs and benefits relative to the present, is often associated with greater engagement in risky behaviors. Incentives such as conditional cash transfers (CCTs) have the potential to counter the effects of high discount rates on health behaviors.

**Methods:**

With data from a randomized trial of a CCT intervention among 434 HIV-positive pregnant women in the Democratic Republic of Congo, we used binomial models to assess interactions between discount rates (measured using a delay-discounting task) and the intervention. The analysis focused on two outcomes: 1) retention in HIV care, and 2) uptake of prevention of mother-to-child transmission (PMTCT) services.

**Results:**

The effect of high discount rates on retention was small, and we did not observe evidence of interaction between high discount rates and CCT on retention. However, our findings suggest that CCT may mitigate the negative effect of high discount rates on uptake of PMTCT services (interaction contrast (IC): 0.18, 95% CI: − 0.09, 0.44).

**Conclusions:**

Our findings provide evidence to support the continued use of small, frequent incentives, to motivate improved uptake of PMTCT services, especially among women exhibiting high rates of temporal discounting.

**Trial registration:**

Clinicaltrials.gov number NCT01838005, April 23, 2013.

**Supplementary Information:**

The online version contains supplementary material available at 10.1186/s12889-021-10499-0.

## Contributions to the literature

- Conditional cash transfer (CCT) interventions demonstrate varying degrees of effectiveness in improving engagement in HIV care. Understanding how these interventions work allow for better adaptation in different contexts, though few studies have explored the underlying mechanisms of CCT.
- We assessed whether a CCT invention involving modest cash incentives works by mitigating the effect of high temporal discount rates on disengagement from care, a major posited mechanism of this type of intervention, among HIV infected pregnant and breastfeeding women in the Democratic Republic of Congo.
- Our findings contribute to gaps in literature surrounding the role of temporal discounting, and its mitigation by CCT, on engagement in HIV care.

## Background

The rapid scale-up of interventions to prevent mother-to-child transmission of HIV over the past decade has led to drastic reductions in the number of new HIV-1 infections among children, which fell by an estimated 35% between 2010 and 2017 [1]. This drop is largely attributed to expansions in access to antiretroviral drugs (ARVs) among pregnant and breastfeeding women. If taken throughout pregnancy and breastfeeding, antiretroviral therapy (ART; a combination of ARVs) can reduce the risk of vertical HIV transmission to approximately 1% [1], compared to 20–25% in the absence of any intervention [2]. Nonetheless, despite broad access to ART, approximately 160,000 children were infected with HIV worldwide in 2018 [3].

The continued occurrence of new HIV infections among children has been attributed to inadequate adherence to ART and poor engagement with the series of steps in care necessary to maximize maternal and infant health outcomes, known as the prevention of mother-to-child transmission (PMTCT) cascade [4–8]. The PMTCT cascade includes attendance at regular clinic visits (i.e., retention in care, at a minimum for ARV medication refills), delivery in a health facility, and testing of the HIV-exposed infant at specific post-partum time points (6 weeks, 9 months, and at breastfeeding cessation).

In many countries with high HIV prevalence, only a limited number of women and their infants complete the full PMTCT cascade [8–10]. Failure to complete the cascade can hinder ART adherence, which can in turn lead to suboptimal viral suppression [11], increased risk of vertical transmission [5], and the development of drug resistance [12, 13]. Further, failure to access adequate antenatal care (including HIV services, as appropriate), or deliver within a health facility, substantially increases the risk of maternal mortality among HIV-infected women [14].

Insight from many disciplines, both within and outside of public health, can inform novel approaches to identify and mitigate the factors contributing to disengagement from the PMTCT cascade. The field of economics, for example, has gained considerable recognition for its ability to help understand choices surrounding HIV prevention and treatment [15, 16]. Specifically, the study of decisions involving tradeoffs between immediate vs. future costs and benefits – referred to as *intertemporal preferences* - provides insight into decision-making processes that lead to certain risky behaviors, including poor retention to care [15–18]. Of key consideration in the study of intertemporal preferences is *temporal discounting,* or the tendency to discount the utility, or value, of future costs and benefits relative to those in the present. High levels of temporal discounting could influence actions surrounding HIV care by causing an individual to value the immediate benefits of disengaging from care (e.g., avoiding stigma, monetary and opportunity cost to attend regular clinic visits, or rude or abusive interactions with clinic staff [19]) over the substantial future benefits of completing the full PMTCT cascade (e.g., PMTCT and disease management). Similarly, individuals may also discount future costs associated with not seeking care today (such as poorer health in the future).

Behavioral interventions using CCT could mitigate the negative impact of high levels of temporal discounting on a variety of health behaviors. CCT involve cash payments to individuals or households on the condition that recipients take certain actions or engage in healthy behaviors [20]. Through the provision of these frequent, small incentives, CCT can enhance the immediate rewards, and reduce the immediate costs, associated with adherence and retention [21]. In doing so, CCT could lead to improved engagement in HIV care, especially among those who exhibit high temporal discounting [15]. To illustrate, Yotebieng et al. found evidence that CCT could improve retention to PMTCT care by mitigating the cost of clinic attendance [22]. In this context, women who exhibit higher temporal discounting may over-weight the immediate costs of transportation to the clinic or, similarly, the mitigation of these immediate costs. If CCT are contingent on engagement in certain health behaviors upon clinic attendance, those with high discounting may then be more likely to engage in these behaviors in order to recoup the losses incurred from transportation. In randomized controlled trials (RCTs) evaluating CCT and other interventions involving conditional economic incentives to improve engagement in HIV care services, however, results demonstrate varying degrees of efficacy [23–30]. Exploring why CCT does or does not work in practice warrants a deeper understanding of how such intervention may affect the mechanisms underlying low engagement HIV care. Nonetheless, few studies to date explore how CCT works to promote retention and engagement in HIV care [31]. None have examined whether the effects of CCTs are moderated by individuals’ intertemporal preferences, even as inordinately high temporal discounting from the present is often cited as one important mechanism of action of CCT in this context [31]. Assessing the modifying role of CCT on the association between intertemporal preferences and risky health behavior could provide insight into how CCT works to improve health outcomes. The objective of the present study is then to examine the impact of CCT on the effect of high temporal discounting on engagement at key points in the PMTCT cascade (i.e., through retention and uptake of available PMTCT services) throughout pregnancy and breastfeeding.

## Methods

We performed a secondary analysis of data from an RCT conducted in Kinshasa, the Democratic Republic of Congo. The parent study’s objective was to assess the effect of a CCT intervention on retention throughout the PMTCT care continuum and uptake of available services among newly-diagnosed HIV-infected pregnant women. Between April 2013 and August 2014, 434 pregnant women were enrolled from 89 maternal and child health clinics throughout Kinshasa. Participants were enrolled at ≤32 weeks of gestation and randomized between 28 and 32 weeks gestation to receive either standard of care or modest and increasing cash incentives (starting at $5 and increasing by $1 each visit), conditional on attending scheduled clinic visits, providing a blood sample for a CD4+ T-cell count, accepting referral for ARVs if referred, delivering in a health facility, and providing a blood sample for infant HIV diagnosis at 6 weeks postpartum. Women who were randomized to the treatment group received their cash incentive at the clinic upon completion of their scheduled appointment (contingent on uptake of PMTCT services), and healthcare providers were blinded to study assignment. A comprehensive description of the parent study methods and results, which showed that the CCT intervention increased retention in HIV care but did not improve ARV adherence or viral suppression among those retained in care, has been published previously [25, 32]. All participants included in the parent study were eligible for this analysis. Reporting of our study was guided by the Strengthening the Reporting of Observational Studies in Epidemiology (STROBE) guidelines.

### Outcomes

We assessed two primary outcomes: 1) retention to HIV care, and 2) complete uptake of all available PMTCT services. Women were classified as retained if they were in HIV care (if they attended their appointment within +/− 4 days of the scheduled date of the appointment) at 6 weeks postpartum, whereas women who were not present in care at 6 weeks postpartum were classified as not retained, regardless of the reasons for not being in care (e.g., poor pregnancy outcome). We determined complete uptake of available PMTCT services based on participants’ completion of all the following conditions: attendance at all scheduled clinic visits (within 5 days of the scheduled date) from randomization through 6 weeks postpartum (including giving birth in a study clinic) and acceptance of all recommended services including providing blood samples for CD4+ T-cell count and dried blood spot sample for DNA PCR testing at 6 weeks.

### Temporal discounting

To assess temporal discounting, participants were told to suppose they had won a prize. Then, they were asked to choose among a series of two hypothetical monetary options for payment, the first being a smaller reward offered immediately, and the second being a larger reward which would be given to the participant after 1 year. This series, known as a delay-discounting task, operated on a gradient, with a steadily greater reward offered in 1 year ($55 today vs. $60 in 1 year, $55 today vs. $80 in 1 year, $55 today vs. $100 in 1 year). The task concluded with the following question for the interviewer: “Did the respondent understand the questions above?” Respondents were excluded from the analysis if the interviewer indicated that they had not understood the task.

The extent of discounting was determined by an individual’s temporal discount rate (referred to hereafter as ‘discount rate’), or the proportional change in the perceived utility of a given reward through time. Responses to the delay-discounting task in this study indicate a participant’s discount rate from the present to 1 year: the choice of the present smaller reward over the increasing larger reward in 1 year reflects a higher and higher discount rate. For example, an individual who chooses $55 today over $60 in 1 year, but not over $80 in 1 year, would exhibit a lower one-year discount rate than an individual who chooses $55 today over $80 in 1 year. Given the distributions of responses to the delay-discounting task, we classified women into two discount groups: the high discount rate group included women who consistently chose the immediate reward of $55 irrespective of the value of future reward. The low discount rate group included women who chose at least one of the larger rewards that were offered in 1 year.

### Covariates

At enrollment in the study, participants were also interviewed using a structured questionnaire to collect information on depression symptoms, health behaviors and various demographic factors. We evaluated depression symptoms using the Patient Health Questionnaire (PHQ-9) [33].. Depression symptoms were dichotomized into low to moderate depressive symptoms (PHQ-9 score of < 15) and severe depressive symptoms (PHQ-9 score of ≥15). A wealth index variable was calculated via a factor analysis of numerous measures related to financial status, including education, household crowding (i.e., proportion of people living in the house per household room), number of beds in a household, cooking fuel type, household water source and ownership for several assets (see Additional file 1). The factor score was then categorized into 3 categories: upper (2 richest quintiles), middle (3rd quintile) and lower (2 poorest quintiles).

In addition to self-reported demographic and behavioral measures, we also assessed disease severity at enrollment by measuring CD4+ T-cell count (i.e., CD4+ T-cells per milliliter of blood). CD4+ T-cell count was dichotomized into ≤350 cells/mL and > 350 cells/mL categories. Self-reported alcohol use (no reported use vs. reported use), age at enrollment (in years) and CCT intervention arm were also included in the analysis.

### Analysis

#### Quantification of the effect of high discount rates on retention and uptake of PMTCT services

Before assessing whether CCT mitigates the effect of high discount rates on retention and uptake of PMTCT services, it was necessary to ascertain whether high discount rates is correlated with these outcomes. We examined the associations between high discount rates with retention and complete uptake of services by comparing the proportion of participants retained in care or with complete uptake of services by 6 weeks postpartum across the two levels of discount rates (high vs. low) using Pearson’s χ^2^ tests. Next, we used unadjusted and adjusted log-binomial risk models to calculate RRs assessing the effect of high discount rates on retention and complete uptake of PMTCT services at 6 weeks postpartum. For the adjusted analysis, we included several potential confounders based on a priori hypotheses and previous research on factors affecting both temporal discounting and retention to care, including disease severity (i.e., CD4+ T-cell count), alcohol use in pregnancy, and age [9, 34, 35]. We then used a backward elimination, change-in-estimate strategy to eliminate hypothesized confounding variables that did not substantially affect the effect estimate (the change-in estimate criterion was 10%) to generate the most parsimonious model that captured the effect of discount rates on retention and complete uptake of services. Further, we included CCT arm and wealth, which were strong predictors of retention and uptake of PMTCT services in previous analyses of this data [32].

#### Interaction analysis

To assess potential interaction of the effect of high discount rates by the CCT intervention, linear risk models were used to estimate the risk difference (RD) measuring the individual and joint effects of the CCT intervention and the high discount rates on each outcome. The likelihood ratio test was used to assess RD modification, and excess risk due to interaction (interaction contrast [IC] and 95% CI) was used to assess for potential effect modification. IC is the difference between the observed RD comparing the doubly-exposed category (high discount rate and receipt of CCT) with the doubly-unexposed category (low discount rate and no CCT or standard of care group), and the expected RD comparing the same two groups (doubly-exposed and doubly-unexposed) assuming additivity (without the interaction term). This difference should be equal to zero in the absence of RD modification. We performed all statistical analyses using SAS version 9.4 (SAS Institute Inc., Cary, NC).

## Results

From the full sample of 434 newly-diagnosed HIV-infected women in the parent study, two women were excluded because the interviewer determined that they had not understood the delay-discounting task, leaving 432 women (99.5% of enrolled participants) in the final analytic sample. Of these remaining women, 332 (76.8%) were retained in care and 263 (60.9%) exhibited complete uptake of services at 6 weeks postpartum. Participants reported a median education level of 10 years (interquartile range (IQR): 8.0–12.0), and the majority of women were married or cohabitating (*n* = 358, 82.9%), and had experienced more than one pregnancy (*n* = 377, 87.3%). Most women (*n* = 374, 86.6%) exhibited high discount rates.

Temporal discounting did not vary by baseline age, alcohol use in pregnancy, wealth or CD4+ T-cell count (see Table 1). A lower proportion of women with a high discount rate exhibited symptoms of severe depression (PHQ-9 score of ≥15) compared to the proportion of women with a low discount rate who had symptoms of severe depression (9.6% vs 25.9%; *p* < 0.001) (see Table 1).

**Table 1:** Characteristics of participants, by strata of temporal discounting (*N* = 432)

	Temporal discounting rate						
	Total (***N*** = 432)		Low rate (***n*** = 58)		High rate (***n*** = 374)		***p***-value^b^
Age: median (IQR)	29 (25–34)		29.5 (27–34)		29 (25–34)		0.34
	*N*	***%***	*N*	***%***	*N*	***%***	
Retention at 6 weeks postpartum^a^							
Retained	158	72.8	24	77.4	134	72.0	0.53
Not retained	59	27.2	7	22.6	52	28.0	
Uptake of PMTCT services^a^							
Complete Uptake	117	53.9	23	74.2	92	49.5	0.01
Incomplete Uptake	100	46.1	8	25.8	94	50.5	
CD4+ T-cell Count							0.66
≤ 350 cells/mL	167	38.7	26	44.8	141	37.7	
> 350 cells/mL	208	48.1	29	50.0	179	47.9	
Missing	57	13.2	3	5.2	54	14.4	
Wealth Index							0.19
Upper	173	40.0	17	29.3	156	41.7	
Middle	86	20.0	13	22.4	73	19.5	
Lower	173	40.0	28	48.3	145	38.8	
Study Group							0.60
Intervention	215	49.8	27	46.6	188	50.3	
Control	217	50.2	31	53.4	186	49.7	
Depression Symptoms							< 0.001
PHQ-9 score < 15	381	88.2	43	74.1	338	90.4	
PHQ-9 score ≥ 15	51	11.8	15	25.9	36	9.6	
Alcohol use in pregnancy							0.14
Yes	108	25.0	19	32.8	89	23.8	
No	324	75.0	39	67.2	285	76.2	

*IQR* Interquartile Range, *CCT* Conditional Cash Transfer, *PHQ-9* Patient Health Questionnaire

^a^Analysis limited to individuals who did not receive the conditional cash transfer intervention (*n* = 217) in order to avoid possible mixing of effects

^b^For continuous variables, *p*-values were calculated using the Wilcoxon Rank Sum test. For categorical variables, p-values were calculated using Pearson’s χ^2^ test. All tests used complete case analysis (i.e., omitting missing observations)

### The effect of discount rates on retention and uptake of PMTCT services

The proportion of participants retained in care at six-weeks postpartum was lower among those with high compared with low discount rates (72.0% vs 77.4%; Table 1), though we did not observe evidence of an association between high discount rates and retention in unadjusted analysis (RR: 0.64; 95% CI:0.32, 1.25; see Table 2). For the multivariable analysis of retention, alcohol use in pregnancy, wealth and severe depression were identified as potential confounders using the change-in-estimate strategy. Adjustment for CCT arm, alcohol use in pregnancy, wealth and severe depression did not alter the association between high discount rates and retention (RR: 0.64; 95% CI: 0.31–1.32; see Table 2).

**Table 2:** Unadjusted and adjusted risk ratios (RR) for the effect of temporal discounting rates on retention and complete uptake of PMTCT services

	Discount rate	RR (95% CI)		Adjusted RR (95% CI)^**a**^	
***Outcomes***					
Retention	High	0.64	(0.32–1.28)	0.64^a^	(0.31–1.32)
	Low	1	(ref)	1	(ref)
Complete uptake of PMTCT services	High	0.63	(0.41–0.95)	0.61^b^	(0.40–0.93)
	Low	1	(ref)	1	(ref)

*CI* Confidence Interval

^a^Adjusted for CCT arm, wealth index, depression symptoms and alcohol use in pregnancy

^b^Adjusted for CCT arm and wealth index

The proportion of participants who exhibited complete uptake of PMTCT services care was considerably lower among those with high compared with low discount rates (49.5% vs 74.2%; Table 1). In unadjusted analysis, women with high discount rates were less likely to exhibit complete uptake of available PMTCT services compared to women with low discount rates (RR: 0.63; 95% CI: 0.41, 0.95; see Table 2). No confounders were identified using the change-in-estimate strategy for uptake of available services, and adjustment for CCT arm and wealth did not meaningfully alter the association between high discount rates and complete uptake of available PMTCT services (RR: 0.61; 95% CI: 0.40, 0.93; see Table 2).

### Modification of the effect of discount rates on retention and uptake of PMTCT services by CCT

Participants who had high discount rates and did not receive CCT had exhibited slightly lower retention risk relative to their counterparts with low discount rates (RD (95% CI): − 0.05 (− 0.24, 0.14) Table 3). We found no or little evidence that receiving CCT modified the effect of high discount rates on retention (IC (95% CI): − 0.08 (− 0.30, 0.14); Table 3). Regarding uptake of PMTCT services, compared to participants with low discount rates who did not receive CCT (i.e., doubly unexposed), those with high discount rates were less likely to exhibit complete uptake of PMTCT services (RD (95% CI): − 0.24 (− 0.44, − 0.04)), and receiving CCT substantially attenuated this negative effect of high discount rates (0.17 (− 0.15, 0.48) Table 3), though these effects were not statistically significant.

**Table 3:** Risk differences (RD) and interaction contrasts (IC) for retention and uptake of PMTCT services at 6 weeks postpartum by strata of temporal discounting rates among 432 newly identified HIV-infected women in Kinshasa, Democratic Republic of Congo

	RD (95% CI)		IC (95% CI)
	Intervention Group	Control Group	
**Retention in Care**			
Discount rate			
High	0.02 (−0.16, 0.20)	−0.05 (− 0.24, 0.14)	−0.08 (− 0.30, 0.14)
Low	0.15 (− 0.04, 0.35)	0 (ref)	
**Uptake of all PMTCT Services**			
Discount rate			
High	−0.07 (− 0.26, 0.11)	−0.24 (− 0.44, − 0.04)	0.17 (− 0.15, 0.48)
Low	0.00 (− 0.27, 0.26)	0 (ref)	

*CI* Confidence Interval, *CCT* Conditional Cash Transfer

## Discussion

Among recently diagnosed HIV-infected women in the Democratic Republic of Congo, we found that complete uptake of recommended PMTCT services was considerably lower among women with high discount rates, even after adjusting for covariates. Further, in alignment with our hypothesis concerning the mechanisms by which CCTs affect health behaviors, we observed evidence suggesting that the negative effects of high discount rates on complete uptake of services were mitigated by a CCT intervention involving small and increasing cash incentives, contingent on engagement in PMTCT care. Though our results suggest a similar antagonist of CCT on the negative effect of discount rates on retention in care, our study did not, however, have enough power to access modification of such a small effect.

Measures of both morbidity and mortality are believed to contribute to high rates of temporal discounting, as they reduce utility and opportunity for consumption in the future, respectively [34–36]. Thus, it was surprising that our analyses revealed no significant association between discounting and several factors that could affect one’s health and survivability, including wealth. One possible explanation for these findings is that, while poverty could lead to high discount rates, high discount rates could positively affect wealth (i.e., inverse causality). Though high levels temporal discounting has often been linked to behaviors that perpetuate the cycle of poverty and ill health among low-income populations [35], discounted utility can – to some extent – be fiscally prudent. The grounds for this prudency are: 1) positive interest rates (i.e., $55 received now would accrue interest and could be worth more than $60 in 1 year), and 2) future uncertainty (i.e., given the possibility of events that may threaten future utility, $55 today carries definite value, whereas the value of $60 in 1 year is uncertain) [37]. Thus, placing more value on immediate rewards relative to those received in 1 year could – to some degree – be a rational adaptive response, particularly in situations of extreme uncertainty.

Another possible explanation for the lack of association between discounting and wealth, may lie in the unique life-situation faced by our study population at enrollment: pregnancy. It could be possible that pregnant women experiencing scarcity may prefer to receive money at a later date, after their delivery, in order to ensure they have adequate resources to support their baby. Though poverty could lead to high discount rates, this effect could be tempered or counteracted by fear for the well-being of their unborn child. It is difficult to assess if this might be occurring, however, without data on parental concern or a comparison group of non-pregnant women.

With regards to the impact of temporal discounting on our key outcomes, the stronger association between discount rates and uptake of available services, relative to retention to care, could suggest that temporal discounting is more closely linked to decision-making upon attendance at clinic appointments, including the acceptance of all proposed services (e.g., provision of blood samples). As with retention, no study to date has assessed the impact of temporal discounting on uptake of PMTCT services, so we are unable to compare our results to other findings. Two studies, however, have assessed the effect of discounting on adherence to ART (i.e., taking the ART medications as prescribed). In a study of HIV-infected adults in Uganda, investigators Linnemayr and Stecher found that individuals with a high discount rate exhibited significantly lower medication adherence than those with a low discount rate [38]. Similarly, in a study of HIV-positive adults on ART in Kenya, Thirumurthy et al. reported lower medication adherence among those with a high discount rate, though this association was not statistically significant, and that high discount rates were associated with significantly higher risk of mortality [39]. Though not directly comparable to our results surrounding uptake of PMTCT services, these previous findings may also suggest that temporal discounting may influence individuals’ decisions surrounding HIV care, beyond attendance at clinic visits.

Acceptance of the necessary testing and treatment, in addition to retaining in care, represent crucial steps within the PMTCT cascade [40]. Uptake of these services may be especially challenging in the Democratic Republic of Congo, as many women may be afraid to publicly or privately acknowledge their diagnosis; HIV is highly stigmatized and is often perceived as a moral failing, thus accepting HIV-specific services (and acknowledging one’s diagnosis in doing so) can have damaging social and personal implications [41, 42]. Retention, by contrast, is measured by attendance at routine clinic visits for prenatal and infant care, and thus does not implicate an acknowledgement of – and reaction to – one’s HIV status. Strong sources of external motivation may then be necessary for many women to overcome their fears surrounding the acknowledgement of their HIV status via uptake of HIV-specific services, beyond retention in care.

One such source of motivation may be the coverage of transport costs upon arrival for routine clinic visits. In a previous study using the same data, Yotebieng et al. found evidence suggesting that CCT improves retention by mitigating the cost of clinic attendance [22]. Among women wishing to attend clinic visits as part of routine prenatal or infant care, but who are unable to do so because of travel difficulties, CCT could provide enough money to cover the cost of transport. Women exhibiting high discount rates from the present may experience a strong impetus to accept HIV-related services, as they are offered immediate monetary incentive for these behaviors and may overweight the value of this incentive. The immediate costs of clinic attendance, too, may be over-weighted among these women, and so they may be more likely to accept HIV-services to recoup their losses. These sources of motivation, rooted in the provision of CCT, could potentially overcome the objections to acceptance HIV-related services among those who are afraid to acknowledge their HIV status.

Notably, CCT is one of several potentially effective strategies to improve engagement in PMTCT care. Many structural support interventions (e.g., integration of routine antenatal care and ART services) and social support interventions (e.g., peer support ‘buddies’ or groups) can improve engagement in HIV and PMTCT care among women in lower- and middle-income countries [43–45]. Tailored interventions, which may involve a combination of structural and social support interventions, could help address the specific needs and multifaceted barriers to care faced by pregnant and breastfeeding women living with HIV [44]. CCT, being relatively low-cost and easy to implement, could be a useful complement to these tailored interventions, especially among women exhibiting high discount rates.

Our findings signify qualitative trends that could provide evidence for our hypothesis; however, they do not reflect definitive findings regarding the mechanisms of this intervention involving CCT. We interpreted our results as indicating potential antagonism between high discount rates and CCT on uptake of services, despite the limited statistical power of this study. Though moderate in size, the parent study was designed to detect the main effects of CCT, not interaction between high discount rates and CCT, and only a small proportion of the study population exhibited low discount rates.

Further, we were unable to directly assess whether participants displayed time-inconsistent (or present biased) preferences, which could be an additional barrier to behaviors involving immediate costs and delayed benefit [46]. Though high discounting from the present could be indicative of present bias, it may also reflect high levels of time-consistent discounting (i.e., general rates of discounting, rather than an inordinate preference for the present) [47, 48]. It should also be noted that our findings reflect the context, population and parameters of this specific CCT intervention, and may not be generalizeable to other interventions involving economic incentives.

There is some disagreement as to the appropriate method to measure temporal discounting, especially within low-income countries. Most measures of discounting were developed for a Western setting, and few studies have evaluated the use of existing measures across different cultural and economic contexts. Some argue that effort tasks (i.e., where individuals are asked to choose between earlier or later activities) should be preferred over monetary tasks (i.e., where individuals are asked to choose between earlier or later amounts of money), especially in low-income settings [49, 50]. The rationale for this argument is that the monetary discount rates in low-income contexts may reflect transient economic needs and not general intertemporal preferences. Recent studies have provided compelling evidence that, for those in poverty whose income is variable and subject to shocks, measures of discounting may capture volatile economic circumstances and not entrenched temporal preferences [51, 52]. As we did not measure discounting levels before and after shifts in economic circumstances, we do not know whether our measures of discounting reflect intrinsic preferences or situational constraints. Nonetheless, our study is among the first to explore any potential mechanisms by which CCT are hypothesized to improve HIV treatment and prevention [53] and high monetary discounting, regardless of cause or permanence, could arguably have similar effects on behavior in the immediate future when monetary rewards are offered, as is the case in our study.

## Conclusions

By identifying the underlying mechanisms of behavior change, and assessing whether a given intervention can influence these mechanisms, public health practitioners can develop more effective strategies to initiate and maintain healthy behaviors (i.e., a mechanisms-focused approach to behavior change research) [54]. Understanding how interventions such as CCT work could identify populations that would most benefit from such an intervention, and permit better adaptation of the intervention into different settings and contexts. From a broader perspective, improving retention and uptake of services among pregnant, HIV-positive women is critical to ensure that PMTCT efforts achieve maximum effectiveness [4]. Our findings provide evidence to support the continued use of small, frequent incentives, to motivate improved uptake of PMTCT services, especially among women exhibiting high discount rates.

## Supplementary Information

**Additional file 1.** Socio-demographic questionnaire.

## Data Availability

The datasets generated and analyzed in the current study are not publicly available in order to maintain confidentiality of study participants but are available from the corresponding author on reasonable request.

## Abbreviations

ART: Antiretroviral Therapy
PMTCT: Prevention of Mother to Child Transmission of HIV
CCT: Conditional Cash Transfer
RCT: Randomized Controlled Trial
RR: Risk Ratio
RD: Risk Difference
CI: Confidence Interval
IC: Interaction Contrast
