# Supplementary material for: Role of temporal discounting in a conditional cash transfer (CCT) intervention to improve engagement in the prevention of mother-to-child transmission (PMTCT) cascade
Source: BMC Public Health. 2021 Mar 10;21:477. doi: 10.1186/s12889-021-10499-0 (PMC7944635; doi:10.1186/s12889-021-10499-0)
Supplement: Supplementary file 1 — Additional file 1. Socio-demographic questionnaire. [file 12889_2021_10499_MOESM1_ESM.docx]

**Additional file 1: Socio-demographic questionnaire**

| **Read: *Now I am going to ask you questions regarding your home, where you have been living since you learned that you were pregnant with this baby. If you have not been living consistently in a single home, think about where you will be living most of the time during this pregnancy.*** | | |
| --- | --- | --- |
| **1** | How long have you lived at your current address? | # of months  *DON’T KNOW = “98” REFUSED = “99* |
| **2** | How many adults (people 18 and older) are living with you at home? |  |
| **3** | How many children under 18 are living with you at home? |  |
| **4** | How many rooms do you have in your house? |  |
| **5** | Do you have electricity at your house? | Yes No |
| **6** | How many of the following, if any, do you have at your house (*check all that apply; do not check if it does not work*): | Beds Radios TVs Mobile phones Refrigerators Bicycles Motorcycles Car |
| **7** | With what do you cook at home? (*check all that apply)* | Wood/Charcoal Gas  Electric Stove |
| **8** | What kind of water do you drink? (Check only one) | Private piped water Communal tap Protected well  Non protected pit  Surface water (stream, river, lake)  Spring/source water Other (*specify*) |
| **9** | Aside from your own housework, have you done any work in the last seven days? | Yes [**SKIP TO 13**]  No |
| **10** | As you know, some women take up jobs for which they are paid in cash or kind. Others sell things, have a small business or work on the family farm or in the family business.  In the last seven days, have you done any of these things or any other work? | Yes [**SKIP TO 13**]  No |
| **11** | Although you did not work in the last seven days, do you have any job or business from which you were absent for leave,  illness, vacation, maternity leave, or any other such reason? | Yes [**SKIP TO 13**]  No |
| **12** | Have you done any work in the last 12 months? | Yes  No [**SKIP TO 17**] |
| **13** | What is your occupation, that is, what kind of work do you mainly do? |  |
| **14** | Do you do this work for a member of your family, for someone else, or are you self-employed? | Family member Someone else Self-employed |
| **15** | Are you paid in cash or kind for this work or are you not paid at all? | Cash only Cash and Kind In kind only  Not Paid [**SKIP TO 17**] |
| **16** | How much were you paid for your work last month or the last month you worked? |  |
| **17** | Has your husband/partner done any work in the last seven days? | Yes [**SKIP TO 21**]  No |
| **18** | As you know, some men take up jobs for which they are paid in cash or kind. Others sell things, have a small business or work on the family farm or in the family business.  In the last seven days, has your husband/partner done any of these things or any other work? | Yes [**SKIP TO 21**]  No |
| **19** | Although your husband/partner did not work in the last seven  days, does he have any job or business from which he was absent for leave, illness, vacation, or any other such reason? | Yes [**SKIP TO 21**]  No |
| **20** | Has your husband/partner done any work in the last 12  months? | Yes  No [**SKIP TO F**] |
| **21** | What is your husband/partner’s occupation, that is, what kind of work does your husband/partner mainly do? |  |
| **22** | Does he do this work for a member of your family, for someone else, or is he self-employed? | For family member For Someone else Self-employed |
| **23** | Is he paid in cash or kind for this work or is he not paid at all? | Cash only Cash and Kind In kind only  Not Paid [**SKIP TO F**] Don’t Know [**SKIP TO F**] |
| **24** | How much was your husband/partner paid last month or the  last month he worked? | Don’t Know |
